# Identification of adrafinil and its main metabolite modafinil in human hair. Self-administration study and interpretation of an authentic case

**DOI:** 10.1080/20961790.2019.1704482

**Published:** 2020-01-29

**Authors:** Alice Ameline, Laurie Gheddar, Jean-Sébastien Raul, Pascal Kintz

**Affiliations:** a Institut de Médecine Légale, Laboratoire de toxicologie, Strasbourg, France; b X-Pertise Consulting, Département de toxicologie, Mittelhausbergen, France

**Keywords:** Forensic sciences, forensic toxicology, adrafinil, modafinil, human hair, UPLC-MS/MS, stimulants

## Abstract

For several years, the misuse of stimulant substances is increasingly observed both in the field of sport, to improve the functions of the body and therefore to be more performant, and also by non-athletes to make life more tolerable on a daily basis. Adrafinil, 2-((diphenylmethyl)sulfinyl)-N-hydroxyacetamide, is a drug designed for the treatment of narcolepsy by promoting an awakened state, and to treat alertness and neurological symptoms in the elderly. It is primarily metabolized *in vivo* to an active form, i.e. modafinil, 2-((diphenylmethyl)sulfinyl)acetamide. The World Anti-Doping Agency (WADA) banned these two drugs in sports in 2004. The authors report an authentic case involving adrafinil and modafinil. The laboratory was requested to test for adrafinil in a hair strand collected from a woman found in possession of vials of adrafinil and suspected of trafficking. A specific method was developed by liquid chromatography tandem mass spectrometry (LC-MS/MS). Unlike modafinil (varying from 6.8 to 13.9 ng/mg), adrafinil was not identified in the strand. The interpretation of the results was difficult because this is the first case describing human hair analysis. In order to be able to interpret the results, a self-administration study was conducted after an oral administration to a volunteer (200 mg) whose beard hair was collected 10 days after administration. The analysis of this specimen highlighted the presence of adrafinil at 0.8 ng/mg and modafinil at 0.5 ng/mg. These results demonstrate the dual identification of both compounds after a single consumption, even after administration of a low dose. According to these results, the analysis of the hair strand from the authentic case does not match with a consumption of adrafinil, in accordance with abuse of modafinil alone. Intelligence considered that this was a trafficking case of adrafinil, with no self-consumption.

## Introduction

The misuse of stimulant substances has been increasingly observed in the past years either by professional athletes to improve the functions of the body and therefore increase performance, as well as by non-athletes to make life more tolerable on a daily basis. Adrafinil, 2-((diphenylmethyl)sulfinyl)-N-hydroxyacetamide, is a drug designed for the treatment of narcolepsy by promoting an awakened state, and to treat alertness and neurological symptoms in the elderly [1]. It was discovered in the late 1970s by Lafon Laboratories. Today, adrafinil is marketed under the name Olmifon^®^ by Cephalon Laboratories, only issued on prescription. It is primarily metabolized *in vivo* to an active form, i.e. modafinil, 2-((diphenylmethyl)sulfinyl)acetamide. Modafinil is also an orally active stimulant used therapeutically in the treatment of narcolepsy and useful in treatment of attention deficit hyperactivity disorders [2]. In 2004, the World Anti-Doping Agency (WADA) banned these two drugs in sports. They are listed section S6a, the non-specified stimulants, on the WADA list of substances prohibited in-competition. In 2017, adrafinil was accounted 0.2% of the substances identified (one case out of 577) while modafinil was accounted 2% of the substances identified (nine cases out of 577) [3].

There is little scientific literature about cases where adrafinil and modafinil were identified in blood and urine, and nothing has been published about their detection in human hair.

The authors report an authentic case involving adrafinil. In order to be able to interpret the results obtained from a hair test, a self-administration study was conducted after an oral administration to a volunteer and subsequent beard collection 10 days after administration.

## Human specimens

### Authentic case report

A 34-year-old escort girl was arrested for adrafinil trafficking. The police found more than 100 small vials of adrafinil in her belongings. She declared that it was for her personal consumption, to have more courage for her difficult job. She denied being a trafficker. The woman was known to the police for having already been convicted for cocaine use. A urine test was performed and revealed the presence of modafinil (around 1 mg/L), the main metabolite of adrafinil, but no trace of the parent molecule. The toxicology laboratory was requested to test for adrafinil in her hair, in order to verify her claims. A hair specimen (12 cm, brown) was collected about 4 weeks after she was arrested, and sent to the laboratory. The specimen was stored at room temperature until analysis.

### Self-administration study

In order (i) to gain information on the detection parameters of adrafinil in hair and (ii) to be able to interpret analytical data, a male subject (58-year-old, 80 kg), one of the authors, ingested 200 mg of adrafinil bought on the Internet and supposed to be at 100% of active material (purity of the powder was verified before the administration). Beard hair was collected 10 days after administration and stored in an envelope at room temperature until analysis.

## Materials and methods

### Chemicals and reagents

**Figure 1.**
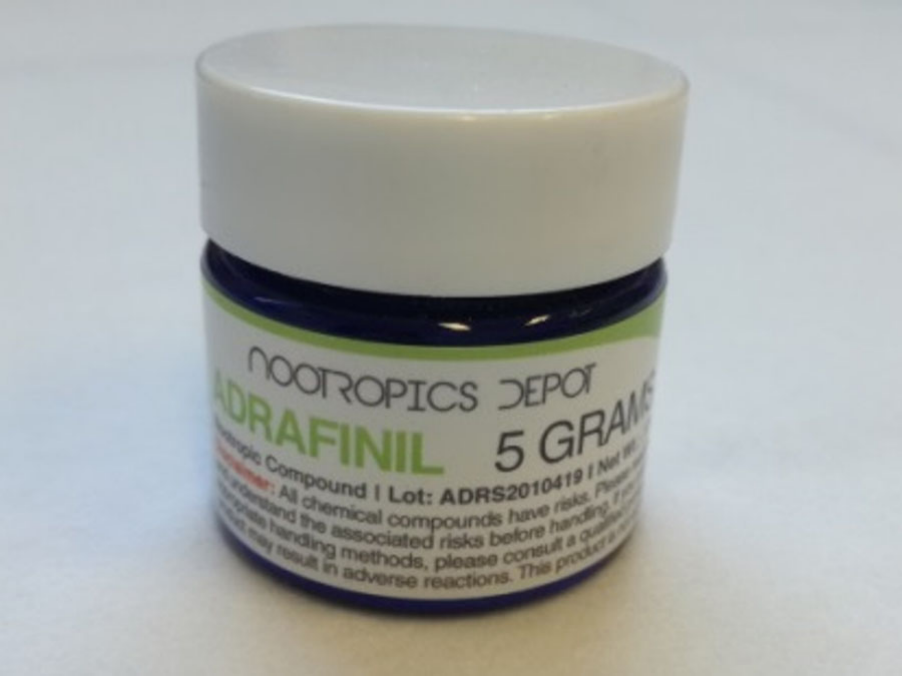
Adrafinil powder, bought on Internet.

Adrafinil (5 g) (Figure 1) was purchased from “Nootropics depot” (Hertfordshire, UK), a stimulating product sales website (https://nootropicsdepot.com), as the drug was not available from standard merchants. This is a common practice in forensic science to obtain reference material. Modafinil, ethyl acetate for liquid chromatography (LC) grade and acetonitrile for LC-MS grade were purchased from Merck (Molsheim, France). Diazepam-d_5_ , used as internal standard, was purchased from LipoMed (Arlersheim, Switzerland). Ammonium formate 99% was provided by Alfa Aesar (Schiltigheim, France). Formic acid 99%–100%, AnalaR NORMAPUR and 36% hydrochloric acid were purchased from VWR Prolabo (Fontenay-sous-Bois, France). Drugs were diluted to appropriate concentrations using methanol.

### Liquid chromatography tandem mass spectrometry (LC-MS/MS) analysis

Adrafinil and its metabolite, modafinil, were extracted from 20 mg decontaminated cut head hair, or cut beard hair, in presence of 20 ng of diazepam-d_5,_ used as internal standard, after overnight incubation in 1 mL technical buffer solution at pH 4.01. Although adrafinil, modafinil and diazepam are chemically very different, the rationale for choosing diazepam-d_5_ is its use during the initial screening of each sample and therefore the possibility of direct confirmation of the extract with a more specific method (i.e. this proposed method). In that way, the same extract can be injected twice, one for screening for general unknown using LC single mass spectrometry, followed by confirmation using LC triple quadrupole mass spectrometry, with the same internal standard. Drugs were extracted in 5 mL of ethyl acetate. After 15 min of agitation at room temperature, centrifugation and evaporation to dryness, the residue was reconstituted in 30 µL of ammonium formate buffer adjusted at pH 3.0. Chromatography was achieved using a Waters Acquity HSS C18 column (150 mm × 2.1 mm × 1.8 µm) maintained at 50 °C in a thermostatically controlled oven. A gradient elution was performed using aqueous formate buffer adjusted to pH 3.0 (mobile phase A) and 0.1% formic acid in acetonitrile (mobile phase B). The flow rate was 0.4 mL/min. The initial gradient was 87% phase A and the final gradient, at 15 min, was 5% phase A. An injection volume of 10 µL was used in all cases. A Xevo TQD triple quadrupole mass spectrometer was used for the detection of the molecule. Ionization was achieved using electrospray in the positive ionization mode (ES+). The following conditions were found to be optimal for the analysis of adrafinil, modafinil and the internal standard: capillary voltage at 1.5 kV; source block temperature at 149 °C; desolvation gas nitrogen heated at 600 °C and delivered at a flow rate of 1 000 L/h. In order to establish appropriate multiple reaction monitoring condition, the cone voltage was adjusted to maximize the intensity of the protonated molecular ion and collision induced dissociated of species was performed. Collision energy was adjusted to optimize the signal for the two most abundant product ions of adrafinil: *m/z* 312.0 > 145.0 (20 eV) and 312.0 > 152.0 (65 eV), modafinil: m/z 296.0 > 129.0 (30 eV) and 296.0 > 152.0 (65 eV) and the most abundant product ion of internal standard m/z 291.9 > 197.8 (32 eV). Transition m/z 312.0 > 145.0 and 296.0 > 129.0 were used to quantify adrafinil and modafinil, respectively. MassLynx 4.1 software (Waters Corporation, Milford, MA, USA) was used for quantification.

### Nuclear magnetic resonance (NMR) analysis

In order to confirm the nature, the purity and the presence of possible other organic substances of the adrafinil powder bought on the Internet, a nuclear magnetic resonance (NMR) analysis was achieved, following method previously published [4,5]. Briefly, the NMR spectra were recorded on AVANCE 300 (Bruker Biospin, Wissembourg, France) operating at 300 MHz equipped with a 5 mm quadruple nucleus probe (QNP) at 295 K. The ^1^H spectra were recorded with 64 scans, 32-K time-domain data points with 4 800 Hz spectral width, an acquisition time of 3.42 s, a relaxation delay of 2 s and a flip angle of 30°. The free induction decays (FIDs) were transformed (0.3 Hz broadening) and the baseline was corrected. 7.5 mg of powder was dissolved in 600 µL of methanol-d_4_ and the ERETIC (Electronic REference To access *In vivo* Concentrations) method was used for the determination of absolute concentrations of adrafinil. ERETIC consisted of a digitally generated Gaussian peak (on single point calibration calibrated using 15 mg caffeine as a reference sample in 600 µL CDCl_3_ and a control point using 10 mg of the same substance in 600 µL CDCl_3_) inserted into the spectrum of a sample by the software after processing the FID. With ERETIC, the value peak area integrals in the spectrum give directly information about the sample purity [6].

## Results and discussion

A specific method to analyze adrafinil and modafinil in human hair by LC-MS/MS was developed. The procedure was validated according ISO 17025 for forensic use. Linearity has been observed for concentrations ranging from 1 to 100 ng/mg, with a correlation coefficient of *r*^2^ = 0.9997 for both compounds. Quality-control samples (1 and 10 ng/mg), analysed in duplicate in 10 independent experimental assays, were used for determination a coefficient of variation for precisions and accuracy. These CVs were lower than 20%. The limit of detection was estimated at 0.1 ng/mg for both compounds, and the lower limit of quantification was the first point of the calibration curve, 1 ng/mg, for both compounds. Under the used chromatographic conditions, there was no interference with the analysis of stimulants (cocaine, amphetamines and NPS) or any extractable endogenous materials present in hair. The matrix effect (<20%) was investigated with spiked adrafinil and modafinil at 10 ng/mg in 10 blank hair samples.

**Figure 2.**
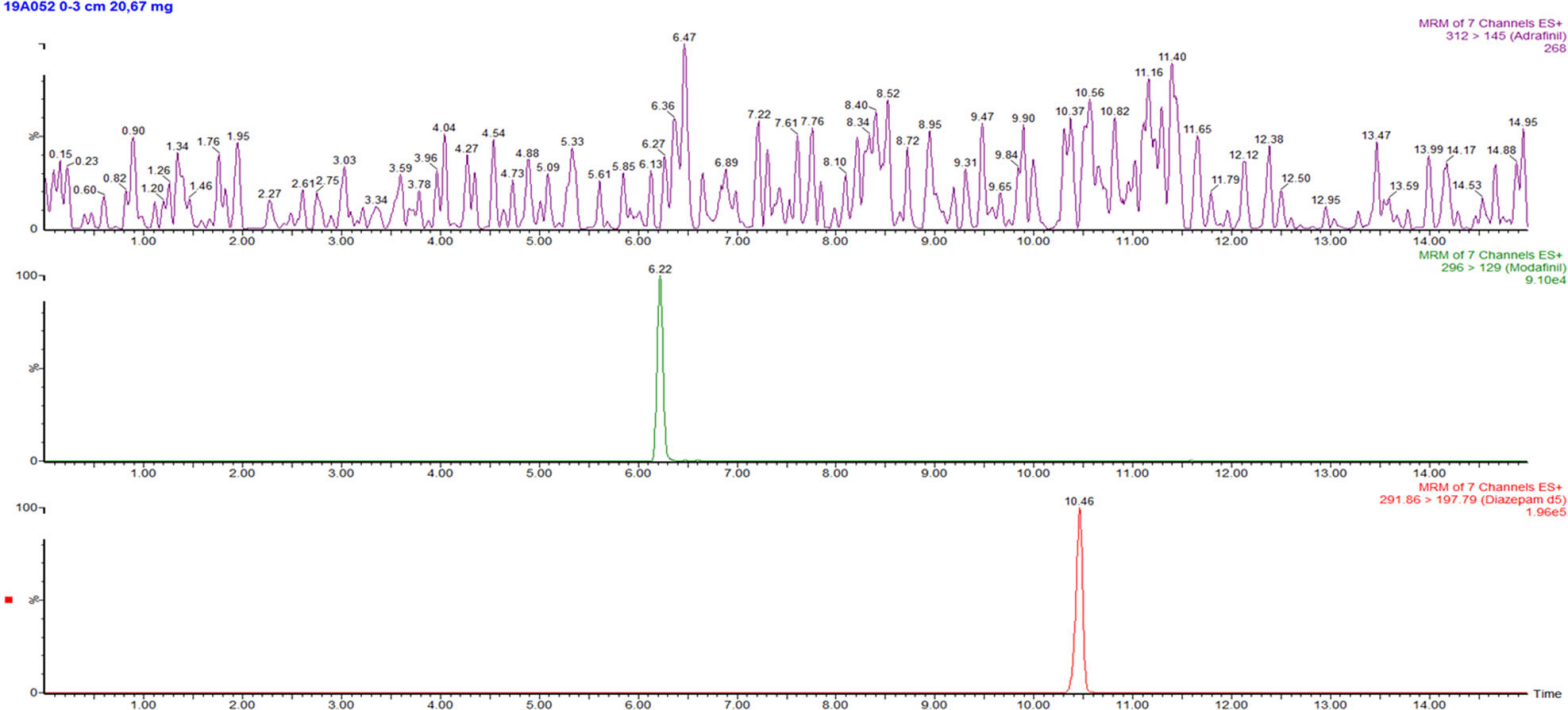
Chromatogram obtained after extraction of the segment 0–3 cm. Adrafinil was not detected and modafinil concentration was 13.7 ng/mg. From top to bottom: quantification transitions for adrafinil (m/z: 312.0 > 145.0), quantification transition for modafinil (m/z: 296.0 > 129.0) and transition for diazepam-d_5_ (m/z: 291.9 > 197.8).

The analysis of adrafinil and modafinil was performed on the hair strand of 12 cm, which was segmented into four segments (0–3 cm, 3–6 cm, 6–9 cm, 9–12 cm) and cut into snippets. The segment corresponding to the period the subject was arrested is the segment 0–3 cm. All the others are representing the period before the arrest, when using the standard 1 cm/month hair growth. The results are presented in Table 1. Chromatograms obtained after extraction of the first segment are presented in Figure 2. Unlike modafinil (from 6.8 to 13.9 ng/mg), adrafinil was not identified in any of the four segments. To our knowledge, this would be the first time in the whole drug testing in hair technology that the parent drug, after consumption, is not detected, while only the metabolite tested positive. It is also possible that the subject did not take adrafinil, in contrast to her claims, but was under long-term abuse of only modafinil. However, the interpretation of these results is difficult because this is the first case involving hair testing for adrafinil and modafinil, with nothing published in the scientific literature.

**Table 1.** Results of the suspect’s hair strand.

	Detection object (ng/mg)	
Segments (cm)	Adrafinil	Modafinil
0–3	ND	13.9
3–6	ND	13.2
6–9	ND	12.3
9–12	ND	6.8

ND: not detected.

In order to gain information on the detection parameters of adrafinil in hair and to be able to interpret the previous analytical results, a self-administration study of adrafinil was performed. The first step was to check the nature of the adrafinil powder purchased on the Internet. No organic impurity was detected using NMR. In addition, the ERETIC method revealed a 94.6% of purity of the powder (Supplementary Figure 1).

After oral administration of 200 mg adrafinil, the overall behaviour of the volunteer was not modified. No physical or behavioural impairment was observed, contrary to what was expected, according to the literature [7,8]. This can be explained by the low dose consumed by the subject, since the active dosages vary between 300 and 1 200 mg/day, according to the patients or abusers.

Ten days after administration, beard hair (0.4 cm) was collected from the volunteer. The analysis of this specimen highlighted the presence of adrafinil at 0.8 ng/mg and modafinil at 0.5 ng/mg. These results demonstrate the dual identification of both compounds after a single administration. This appears as the first identification of adrafinil and modafinil in human hair.

The results obtained during the self-administration study allowed to interpret the findings of the hair test from the authentic case. According to this study, when consuming adrafinil, even after a single consumption at low dose consumed, both adrafinil and modafinil are detectable in hair. Therefore, the results obtained for the analysis of the hair strand from the authentic case do not match with a long-term consumption of adrafinil, as no adrafinil was identify. In this way, it appears that the woman was not a regular consumer of adrafinil, but of modafinil, a well-known stimulant. Trafficking offence was considered as obvious.

## Conclusion

At the date, there are no data in the scientific literature reporting identification of adrafinil in human hair. Secure self-administration studies can be very useful to interpret results of single case. A highly powerful tool, such as NMR has the pre-requisite for such studies, when the chemicals cannot be obtained from reputable suppliers.

In this paper, the authors established the parameters of detection of adrafinil after a single consumption at a low dose. It appears the adrafinil and modafinil are detectable in the hair, with concentrations in the ng/mg range. LC-MS/MS was found as the suitable tool to identify the drugs. This study made it possible to interpret the results of a real case and to demonstrate repetitive consumption of modafinil, and not of adrafinil. As adrafinil is converted into modafinil in urine, the single identification of modafinil in the matrix cannot allow to discriminate the nature of the chemical which was used. Hair, at the opposite, can propose to achieve the definitive identification.

## Supplementary Material

Supplemental Material
