# Supplementary figures and images for: Identification of adrafinil and its main metabolite modafinil in human hair. Self-administration study and interpretation of an authentic case
Source: Forensic Sci Res. 2020 Jan 29;5(4):322–6. doi: 10.1080/20961790.2019.1704482 (PMC7782130; doi:10.1080/20961790.2019.1704482)

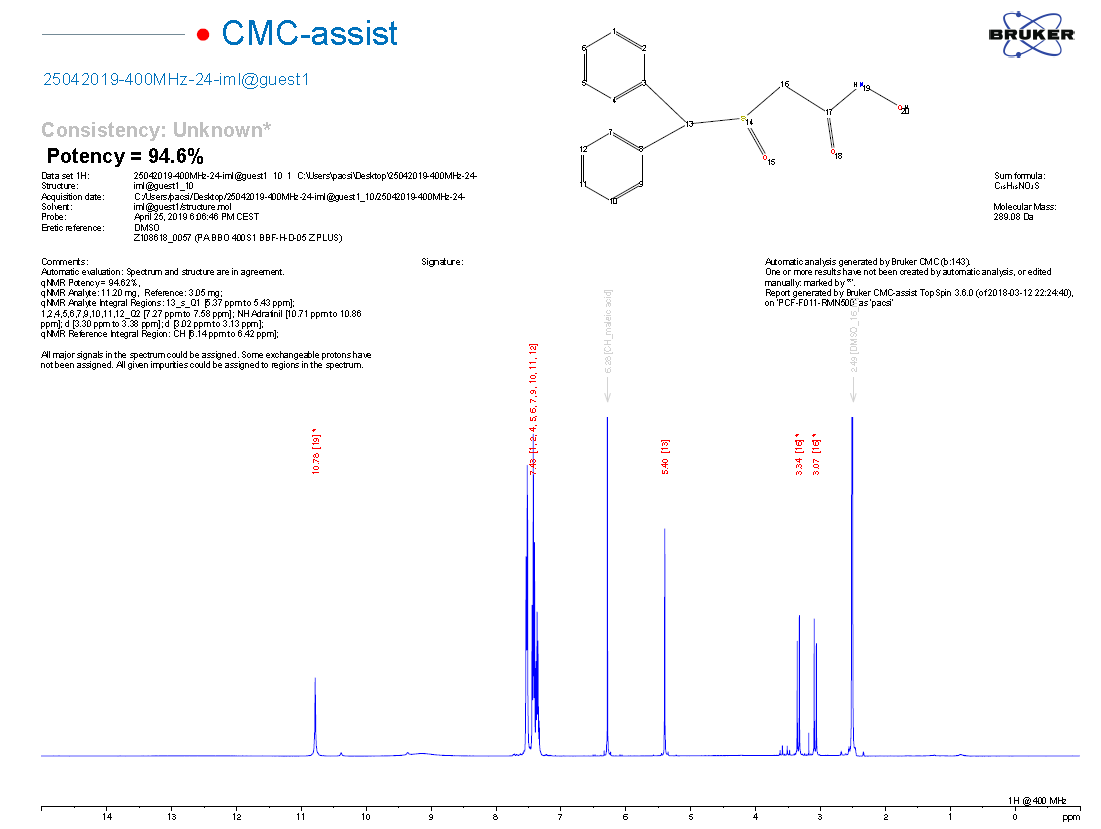

Supplement: Supplemental Material [file TFSR_A_1704482_SM3341.png]
